# Study on Microstructure and Mechanical Properties of TC4/AZ31 Magnesium Matrix Nanocomposites

**DOI:** 10.3390/ma16031139

**Published:** 2023-01-29

**Authors:** Yong Chen, Yuan Yao, Shengli Han, Xiaowei Feng, Tiegang Luo, Kaihong Zheng

**Affiliations:** 1 College of Mechanical Engineering, University of South China, Hengyang 421101, China; 2 Guangdong Provincial Key Laboratory of Metal Toughening Technology and Application, Institute of New Materials, Guangdong Academy of Sciences, National Engineering Research Center of Powder Metallurgy of Titanium & Rare Metals, Guangzhou 510650, China

**Keywords:** magnesium alloy, nano-powder, TC4 particle, microstructure, mechanical properties

## Abstract

In the field of metal matrix composites, it is a great challenge to improve the strength and elongation of magnesium matrix composites simultaneously. In this work, xTC4/AZ31 (x = 0.5, 1, 1.5 wt.%) composites were fabricated by spark plasma sintering (SPS) followed by hot extrusion. Scanning electron microscopy (SEM) showed that nano-TC4 (Ti-6Al-4V) was well dispersed in the AZ31 matrix. We studied the microstructure evolution and tensile properties of the composites, and analyzed the strengthening mechanism of nano-TC4 on magnesium matrix composites. The results showed that magnesium matrix composites with 1 wt.%TC4 had good comprehensive properties; compared with the AZ31 matrix, the yield strength (YS) was increased by 20.4%, from 162 MPa to 195 MPa; the ultimate tensile strength (UTS) was increased by 11.7%, from 274 MPa to 306 MPa, and the failure strain (FS) was increased by 21.1%, from 7.6% to 9.2%. The improvement in strength was mainly due to grain refinement and good interfacial bonding between nano-TC4 and the Mg matrix. The increase in elongation was the result of grain refinement and a weakened texture.

## 1. Introduction

Magnesium alloy is currently the lightest engineering structural material; because of its high specific strength, high specific stiffness, good damping performance, good thermal conductivity, easy recycling, and other characteristics, it is widely used in electronic products, automobiles, aerospace, and other fields [1,2]. However, due to the hexagonal close-packed (HCP) structure of the magnesium matrix, its plasticity is poor, which restricts its wide application [3,4]. Therefore, it is necessary to obtain magnesium matrix composites with other materials through material design to overcome the above defects [5,6]. When using micrometer or larger-scale reinforcement, the strength of the composites is increased at the same time as the plasticity is adversely affected [7,8]. Some studies [9] have shown that if the size of the reinforcement is reduced to the nanometer scale and it has uniform dispersion, the strength of magnesium matrix composites can be improved while the plasticity is maintained. 

It has been widely confirmed that the selection of reinforcing materials also has an important effect on the final properties of composites [10,11]. In recent years, many scholars have developed a variety of composites by adding SiC [12], Al_2_O_3_ [13], B_4_C [14], and other ceramic particles into the magnesium matrix; due to the large differences in the thermal expansion coefficient and elastic modulus between the ceramic reinforcement and magnesium matrix, the addition of these nanoparticles can improve the strength of magnesium but adversely affect the plasticity of magnesium [15,16]. 

Ti and its alloys have good fracture toughness and high specific strength and have similar crystal structures to magnesium at room temperature, which is conducive to the compatibility between Ti and Mg [17]. Recent studies have shown that Ti and its alloys can improve the strength and ductility of magnesium matrix composites as reinforcement particles [18]. Dinaharan et al. [19] prepared Ti/AZ31 composites using friction stir processing; the YS and UTS were improved while maintaining plasticity. Wang et al. [20] studied the effect of hot extrusion on TC4/AZ91 composites; after hot extrusion, the grain size of the composite was refined, and the YS, UTS, and FS of the composite were increased simultaneously. Therefore, Ti and its alloy particles as reinforcements have great potential in the preparation of magnesium matrix composites.

At present, magnesium matrix composites are often prepared by stir casting and powder metallurgy methods. There are some problems in the stir casting method, such as serious settlement and the uneven distribution of reinforced particles [21]. The powder metallurgy method can distribute the reinforced particles evenly in the Mg matrix [22]. To further improve the mechanical properties of magnesium matrix composites, secondary processing techniques such as hot extrusion have been widely applied to magnesium matrix composites prepared by powder metallurgy [20]. Therefore, magnesium matrix composites prepared by powder metallurgy are usually hot-extruded to obtain the expected mechanical properties.

In this study, we analyzed the microstructures and mechanical properties of TC4/AZ31 composites fabricated by SPS followed by hot extrusion. The effects of nano-TC4 on the microstructure and mechanical properties of the AZ31 alloy were discussed.

## 2. Experimental Procedure 

### 2.1. Fabrication of the Composites

**Figure 1:**
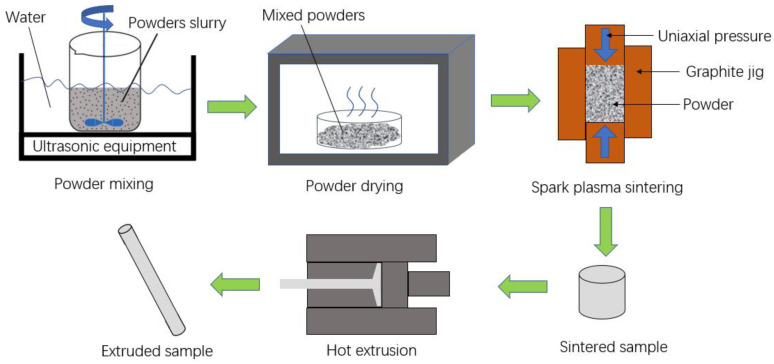
Schematic diagram of TC4/AZ31 composite preparation process.

**Figure 2:**
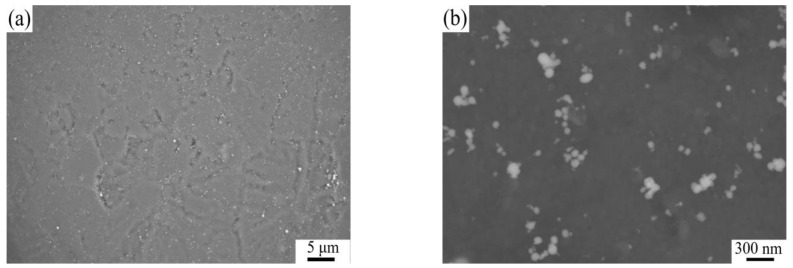
Low-magnification SEM (**a**) and high-magnification SEM (**b**) of 1 wt.%TC4 nanoparticles’ dispersion on the surface of magnesium powder particles.

**Figure 3:**
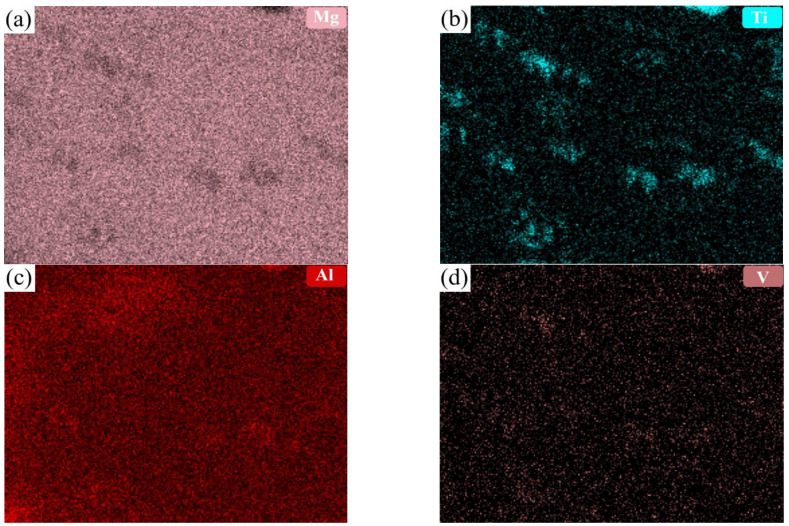
X-ray maps of (**a**) Mg, (**b**) Ti, (**c**) Al, and (**d**) V in Figure 2b.

**Figure 4:**
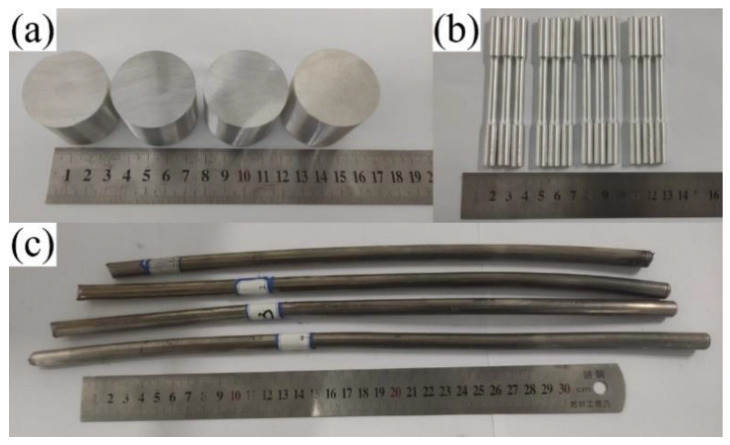
Experimental samples of (**a**) sintered samples, (**b**) tensile test samples obtained by mechanical processing of extruded samples, and (**c**) extruded samples.

AZ31 powders were purchased from Tangshan WEIHAO Magnesium Powder Co., Ltd., China, and the chemical composition is shown in Table 1. TC4 powders were purchased from Guangdong YInna Technology Co., Ltd., China. The particle size of the AZ31 and TC4 powders was 55 μm and 60 nm, respectively. The fabrication procedure is shown in Figure 1. Preparation process of composites: Nano-TC4 was added into acetone solution, ultrasonic dispersion was carried out with a KQ-100E ultrasonic cleaner for 20 min, and we then added the AZ31 powder into the mixture; the mixture continued to be ultrasonicated for 10 min and stirred with an electric mixer at 100 r/min. Finally, we placed the mixture into an electric blast oven for 50 ℃ until the mixture became dry. The dispersion of 1 wt.%TC4 nanoparticles on the surface of magnesium powder particles is shown in Figure 2. Figure 3 shows the X-ray maps of Figure 2b; nano-TC4 was dispersed uniformly and no obvious agglomeration was found. The dried composite material powder was loaded into a graphite mold with an inner diameter of 40 mm and sintered by SPS. The sample is shown in Figure 4a, and the following sintering parameters were set: sintering pressure 40 MPa, heating rate 100 ℃/min, sintering temperature 500 ℃ and holding for 3 min. The sintered sample was a cylinder with a diameter of 40 mm and a height of 30 to 40 mm. We placed the sintered sample into the hot extrusion equipment for processing, and the specific parameters of the hot extrusion process were preheating at 400 ℃ for 30 min, followed by extrusion at 400 ℃, and the extruded samples were air-cooled to room temperature, with an extrusion ratio of 16:1 and extrusion rate of 6 mm/s. After removing the extrusion residue, the extruded samples were round rods with a diameter of 10 mm and a length of 350 to 470 mm; a sample is shown in Figure 4c. 

### 2.2. Microstructure Characterization

Using scanning electron microscopy (SEM; GeminiSEM300), an X-ray diffractometer (XRD; Rigaku SmartLab), and electron backscatter diffraction (EBSD; GeminiSEM300), we studied the microstructure of as-extruded TC4/AZ31 composites. XRD and SEM microstructure observation were performed in the center of the sample perpendicular to the extrusion direction, while EBSD microstructure observation was performed in the center of the sample parallel to the extrusion direction. EBSD samples were subjected to grinding–polishing and then electropolishing with AC2 (800 mL ethanol + 100 mL propanol + 18.5 mL distilled water + 10 g hydroxyquinoline + 75 g citric acid + 41.5 g sodium thiocyanate + 15 mL perchloric acid). SEM and XRD samples were prepared by grinding and polishing. Working parameters of XRD were as follows: using Cu-Kα ray, working voltage 45 kV, working current 0.2 A, scanning rate 2°/min, scanning angle 10° to 80°.

### 2.3. Mechanical Property Testing

**Figure 5:**
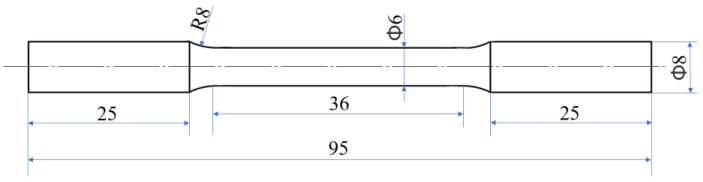
The specific shape and size of tensile test samples.

All tensile specimens were machined parallel to the extrusion direction; the tensile test samples are shown in Figure 4b, and the specific shape and size of tensile test samples are shown in Figure 5. The tensile test of the TC4/AZ31 composites at room temperature was carried out on a universal test machine (CMT5105). To ensure the accuracy of the experimental data, three samples were used for each material, using the GB/T228.1-2021 standard and under the same conditions to obtain the average tensile data (YS, UTS, and FS) of each set of experiments. 

## 3. Results and Discussion

### 3.1. X-ray Diffraction

**Figure 6:**
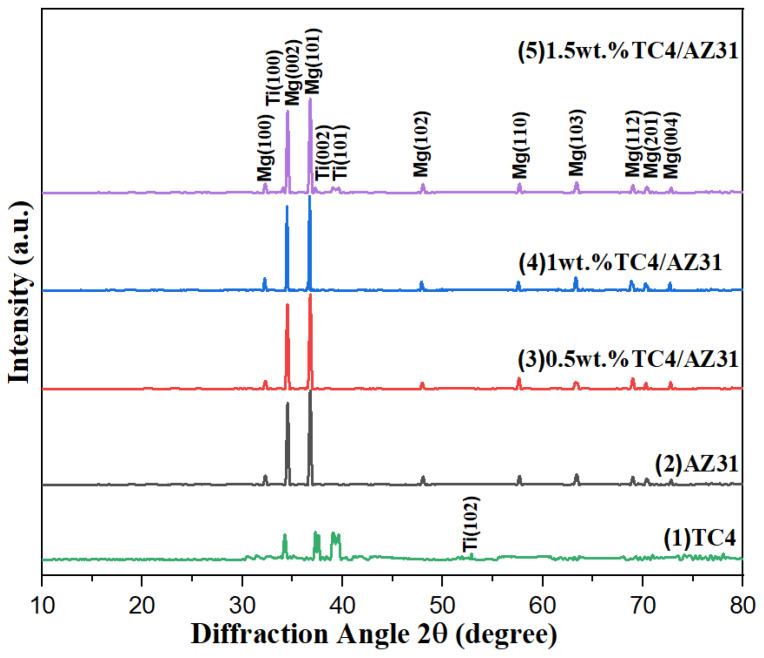
XRD patterns of composites.

Figure 6 shows the X-ray diffraction patterns of TC4, AZ31, 0.5 wt.%TC4/AZ31, 1 wt.%TC4/AZ31, and 1.5 wt.%TC4/AZ31 composites. It is obvious from the figure that the peaks of TC4 appear at 2θ equal to 34.26°, 37.62°, 39.66°, and 52.88°. Different phases of magnesium can be observed at 2θ equal to 32.28°, 34.52°, 36.78°, 48.06°, 57.68°, 63.38°, 69.02°, 70.38°, and 72.82°; compared with the JCPDS of magnesium, the overall peak shifted to the right by approximately 0.2°, but this did not affect the results of the experiment. The addition of nano-TC4 produced new peaks at 2θ equal to 34.08°, 37.28°, and 39.62°, which confirmed the presence of nano-TC4 in the composites. The diffraction peak of TC4/AZ31 composites did not shift with the increase in TC4 content; this was due to the low content and small size of nano-TC4 particles, and only a weak titanium peak appeared in the XRD pattern of the composites.

### 3.2. Microstructure Characterization

**Figure 7:**
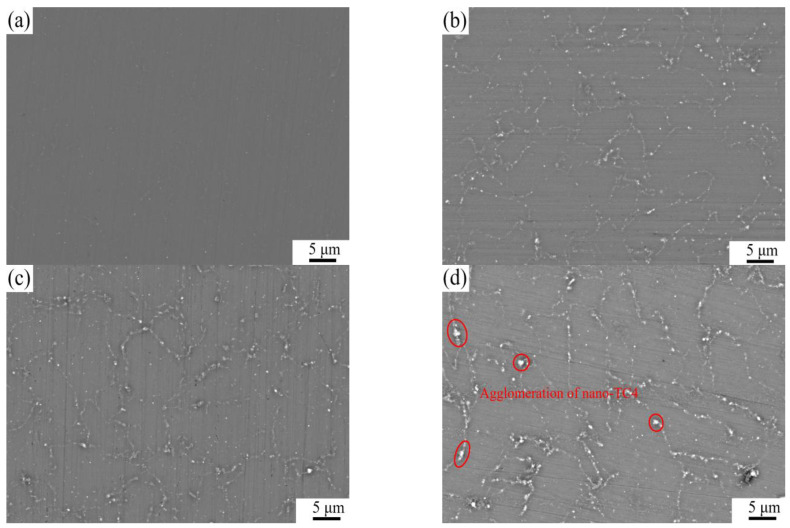
SEM microstructures of (**a**) AZ31, (**b**) 0.5 wt.%TC4/AZ31, (**c**) 1 wt.%TC4/AZ31, and (**d**) 1.5 wt.%TC4/AZ31 as-extruded composites.

Figure 7 shows the SEM microstructures of as-extruded TC4/AZ31 composites. In Figure 7b,c, nano-TC4 particles are uniformly dispersed in the AZ31 matrix. However, in the 1.5 wt.%TC4/AZ31 composite, nano-TC4 aggregates at the boundaries of particles, as shown in Figure 7d. In the process of material preparation, the interaction between the nanoparticles is inevitable. The nanoparticles have huge surface energy and the spacing between the particles is extremely short [23]; under these circumstances, the weaker forces, such as van der Waals forces, electrostatic attraction, and capillary forces, become more and more prominent, which leads to the agglomeration of TC4 nanoparticles in the composites [24]. The agglomeration of nano-TC4 not only hinders the grain refinement of the magnesium matrix but also tends to produce defects in the deformation process, which ultimately leads to the deterioration of the mechanical properties. 

**Figure 8:**
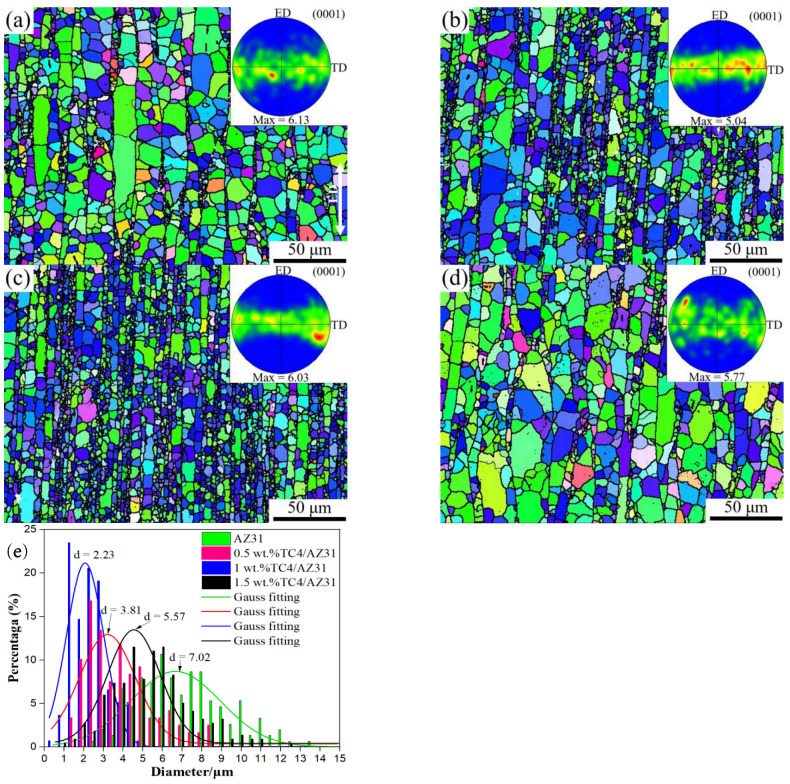
Crystal orientation mapping and macrotexture of (**a**) AZ31, (**b**) 0.5 wt.%TC4/AZ31, (**c**) 1 wt.%TC4/AZ31, and (**d**) 1.5 wt.%TC4/AZ31 as-extruded composites; (**e**) grain size distribution statistics.

Figure 8 shows the crystal orientation mapping and macrotexture of the as-extruded TC4/AZ31 composites. As can be seen from Figure 8, the grain distribution of the matrix and composite material is uniform, and the grain size of the composite material is decreased after the addition of nano-TC4 compared with the matrix. Compared with the AZ31 matrix, the average grain size of 1 wt.%TC4/AZ31 composites was refined from 7.02 μm to 2.23 μm. However, with the further increase in nano-TC4 content, the grain size of 1.5 wt.%TC4/AZ31 composites increased to 5. 57 μm, which was associated with the agglomeration of nano-TC4. The grain size became finer near the reinforcement at the boundary of the magnesium particle; this indicates that nano-TC4 promotes the dynamic recrystallization (DRX) of the composites during the hot extrusion process. Since magnesium is easy to deform and TC4 particles are not easy to deform, misfit stress occurs between the magnesium matrix and reinforcement particles in the process of hot extrusion [25]. This mismatch results in a strain gradient in the magnesium matrix near the reinforcement particles, resulting in an increase in dislocation density and a larger directional derivative [26]. 

The polar density points of the composite pole figure are mainly distributed along the direction perpendicular to the extrusion direction (ED), indicating that the base plane of most grains in the composite is parallel to the extrusion direction, and the texture type is the base fiber texture. With the increase in nano-TC4 content, the maximum texture intensity of the composite decreased from 6.13 for the AZ31 alloy to 5.04 for the TC4/AZ31 composites; this means that the orientation of grains is more random in TC4-containing composites [27]. Randomly oriented grains can coordinate deformation, which is beneficial to improving the plasticity of composites.

**Figure 9:**
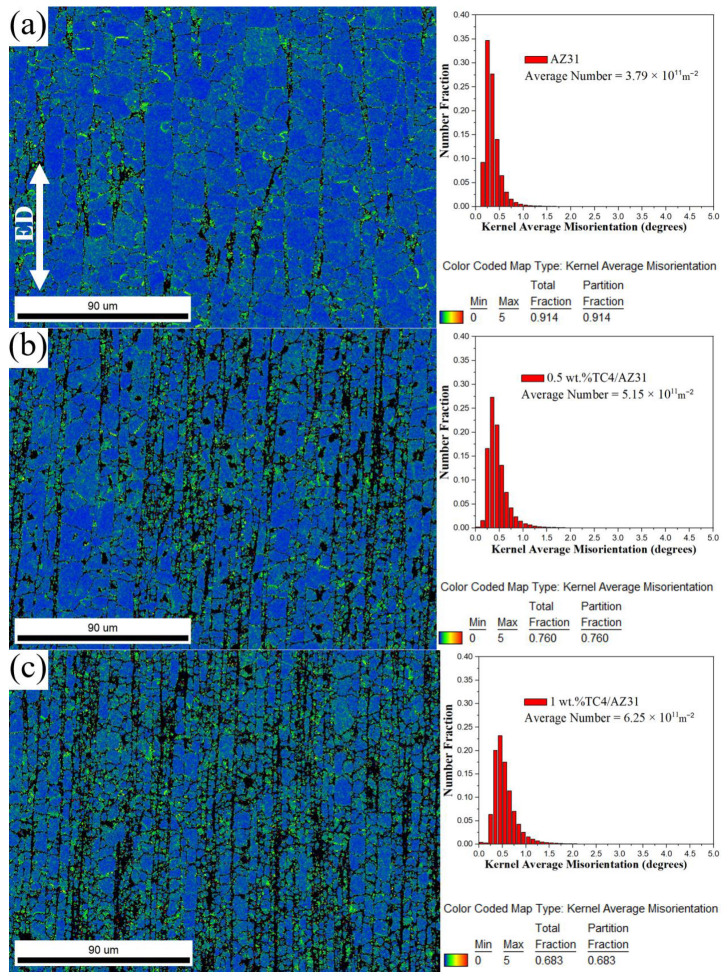

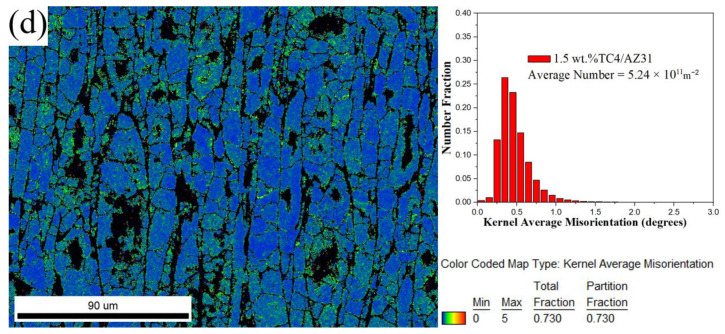
KAM map and KAM value distribution chart of (**a**) AZ31, (**b**) 0.5 wt.%TC4/AZ31, (**c**) 1 wt.%TC4/AZ31, and (**d**) 1.5 wt.%TC4/AZ31 as-extruded composites.

Figure 9 shows the KAM map and KAM value distribution chart of as-extruded TC4/AZ31 composites. To study the evolution of the dislocation density, the kernel average misorientation (KAM) method was used [28]. Using EBSD orientation data to determine the local misorientation angle, we studied the variation in local crystal orientation on the surfaces of composites with different TC4 content. As shown in Figure 9c, the dislocation is clustered around the reinforcement at the boundary of the magnesium particle, which confirms that the misfit stress occurs between the magnesium matrix and the reinforcement particles, leading to the increase in dislocation density in this location. With the increase in nano-TC4 content, the density of geometrically necessary dislocation (GND) increases from 3.79 × 10^11^ m^−2^ for the AZ31 alloy to 6.25 × 10^11^ m^−2^ for the 1 wt.%TC4/AZ31 composites; the higher the dislocation density, the more dislocations need to slip simultaneously per unit area, and this increases the force required for the composite to deform, which has a positive effect on the improvement in the strength of the composite material [29]. However, with the further increase in nano-TC4 content, the GND decreases to 5.24 × 10^11^ m^−2^ for 1.5 wt.%TC4/AZ31 composites, which is mainly caused by the agglomeration of nano-TC4 particles.

**Figure 10:**
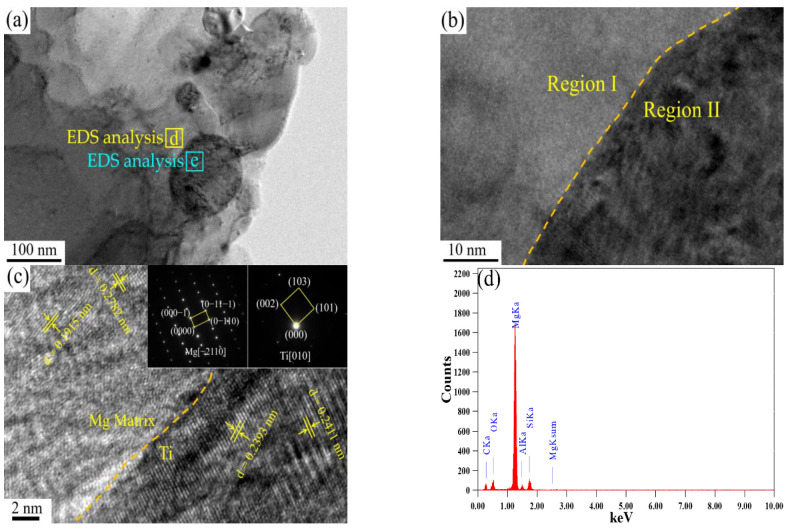

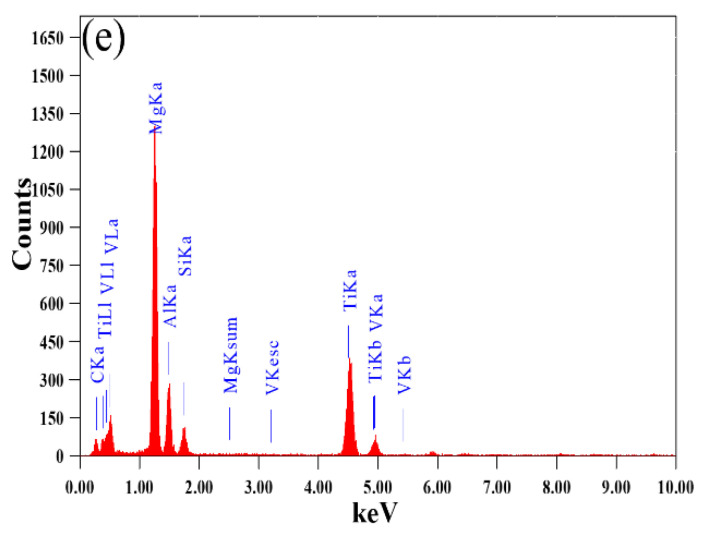
Interfacial structure of as-extruded 1 wt.%TC4/AZ31 composite: (**a**) TEM image; (**b**) HRTEM image; (**c**) Ti/Mg interface; (**d**) EDS analysis in position d; (**e**) EDS analysis in position e.

Figure 10 shows the interface structure of the as-extruded 1 wt.%TC4/AZ31 composite. The interface analysis between TC4 and the magnesium matrix is shown in Figure 10c; by measuring its lattice fringes and analyzing the diffraction spots, region I and region II were determined to be Mg and Ti, respectively. Figure 10d,e show the EDS analysis of regions d and e in Figure 10a; its results show that region d is the AZ31 matrix, and region e is the inclusion of the magnesium matrix and TC4. Ye et al. [30] obtained 6 wt.%Ti/AZ31 composites by smelting at 630 ℃ and found TiAl and Al_2_Ti at the interface of magnesium and titanium, which had a positive impact on the interface bonding strength. Luo et al. [31] obtained 5 wt.%TC4/AZ91 composites by powder metallurgy and found Al_3_Ti at the interface of magnesium and titanium, which had a positive impact on the mechanical properties. In this study, no other phases were found at the interface between TC4 and the magnesium matrix, which was related to the low sintering temperature and short sintering time of the composite.

### 3.3. Tensile Test

**Figure 11:**
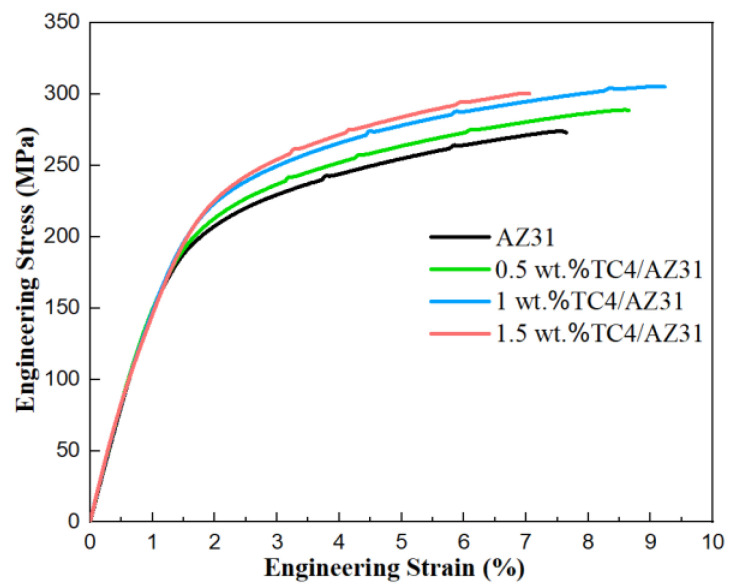
Stress–strain curves of as-extruded TC4/AZ31 composites in tensile tests.

Figure 11 shows the tensile properties of the TC4/AZ31 composites at room temperature. The YS, UTS, and FS of the AZ31 alloy are 162 MPa, 274 MPa, and 7.6%, respectively. The YS, UTS, and FS of the 1 wt.%TC4/AZ31 composite are 195 MPa, 306 MPa, and 9.2%, respectively, which are 20.4%, 11.7%, and 21.1% higher than those of the AZ31 alloy. The 1 wt.%TC4/AZ31 composite realized the simultaneous improvement of YS, UTS, and FS. However, with the further increase in nano-TC4 content, the UTS and FS of 1.5 wt.%TC4/AZ31 composites tended to decrease. To obtain a comparison with this work, statistical methods were used to calculate the mean and variance of the experimental data for each group [32]; the data for some other particle-reinforced magnesium matrixes [30,33,34] are included in Table 2. The UTS and FS of the TC4/AZ31 composites are higher than those of most Mg composites reinforced by ceramic particles. 

The improvement in the tensile strength of the TC4/AZ31 composite is mainly due to the high strength of TC4, the refined microstructure, and the good interface bonding between nano-TC4 and the Mg matrix. Therefore, the strengthening mechanism of composites can be summarized as grain refinement, the dislocation of different coefficients of thermal expansion (CTE), Orowan strengthening, and load transfer from the Mg matrix to TC4 particles.

With the addition of nano-TC4, the grain size of the composite becomes smaller, and the improvement in the YS of the composite by grain refinement can be expressed as [35]

(1) $${\Delta \sigma }_{\mathrm{GR}}=K\left(d_{c}^{-\frac{1}{2}}-d_{m}^{-\frac{1}{2}}\right),$$

where K is the Hall–Petch coefficient of the Mg matrix alloy, and d_c_ and d_m_ are the average grain sizes of composites and the Mg matrix, respectively.

The thermal expansion coefficients of Mg and TC4 are 25 × 10^−6^ K^−1^ and 7.9 × 10^−6^ K^−1^, respectively; the thermal expansion coefficient between the Mg matrix and TC4 particles is strongly mismatched, and this results in a large number of GND at the interface of the Mg matrix and TC4 particles. These dislocations lead to the improvement in the strength of TC4/AZ31 composites. The contribution of the thermal expansion coefficient mismatch in YS can be described by the following equation [36]:

(2) $${\Delta \sigma }_{\mathrm{CTE}}=\sqrt{3}\beta G_{m}b\sqrt{\frac{12\Delta T\Delta CV_{p}}{\left(1-V_{p}\right)bd_{p}}}$$

where β is the geometric constant value. G_m_ and b are the shear moduli and the Burgers vector of the Mg matrix, respectively. ΔC is the difference in CTE between TC4 and the Mg matrix. ΔT represents the change between the extrusion temperature and room temperature. V_p_ represents the volume fraction of TC4 particles. d_p_ represents the size of the TC4 particle. 

According to the Orowan mechanism, due to the distribution of nanoparticles, the movement of dislocations is hindered and the strength of materials is improved [37]. The dislocation movement encountered the reinforcement particles and generated reverse stress, which hindered the dislocation migration. Therefore, a dislocation loop was formed around the reinforcement particles, resulting in the improvement in the YS of the material [38]. Orowan enhancement can be calculated using the following equation [39]:

(3) $${\Delta \sigma }_{\mathrm{Orowan}}=\frac{0.13G_{m}b}{\lambda }\ln\frac{d_{P}}{2b},$$

where λ is the particle spacing, calculated using the following equation [40]:

(4) $$\lambda \approx d_{p}\left[{\left(\frac{1}{{2V}_{p}}\right)}^{\frac{1}{3}}-1\right].$$

In the process of extrusion, the interface bonding determines whether the load can be transferred from the soft matrix to the hard reinforcement. As can be seen from Figure 7, the nano-TC4 particles are dispersed in the magnesium matrix with good interfacial bonding; this indicates that the matrix can effectively transfer the load to the reinforcement, and the strength of the material can be improved. The increase in YS caused by load transfer can be expressed by the following equation [41]:

(5) $${\Delta \sigma }_{\mathrm{LT}}=\frac{V_{p}\sigma_{m}}{2},$$

where Δσ_LT_ is the increase in YS, and σ_m_ is the YS of the Mg matrix.

Based on the parameter values listed in Table 3, the theoretical YS of composites can be calculated by the following formula:

(6) $$\sigma_{\mathrm{theoretical}}=\sigma_{m}+{\Delta \sigma }_{\mathrm{GR}}+{\Delta \sigma }_{\mathrm{LT}}+{\Delta \sigma }_{\mathrm{CTE}}+{\Delta \sigma }_{\mathrm{Orowan}}$$

**Figure 12:**
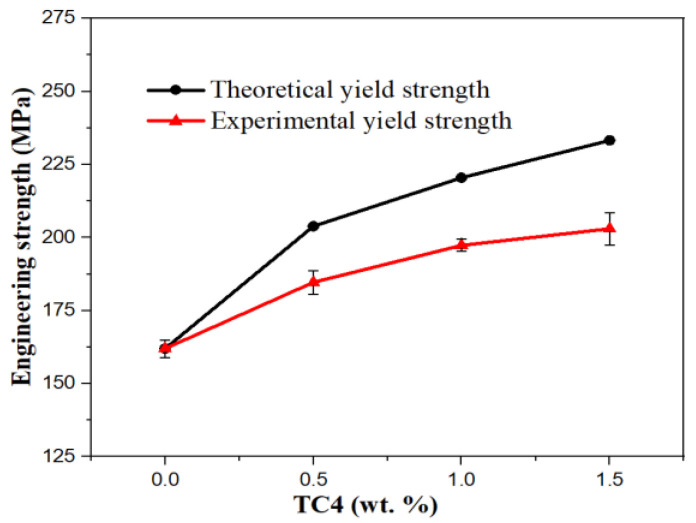
Comparison of theoretical and experimental YS of composites.

The theoretical YS of the composite is shown in Figure 12; the theoretical and experimental YS of TC4/AZ31 composites increase with the increase in nano-TC4 content. The experimental YS is always smaller than the theoretical YS, and the gap becomes wider with the increase in nano-TC4 content. This is because the theoretical calculation is based on the completely uniform dispersion of nano-TC4 in the Mg matrix. In practice, nano-TC4 forms aggregates in the matrix, and the more TC4 content there is, the easier it is to form aggregates. Therefore, the experimental YS is smaller than the theoretical YS, and the gap increases with the increase in TC4 content.

### 3.4. Fracture Surface Analysis 

**Figure 13:**
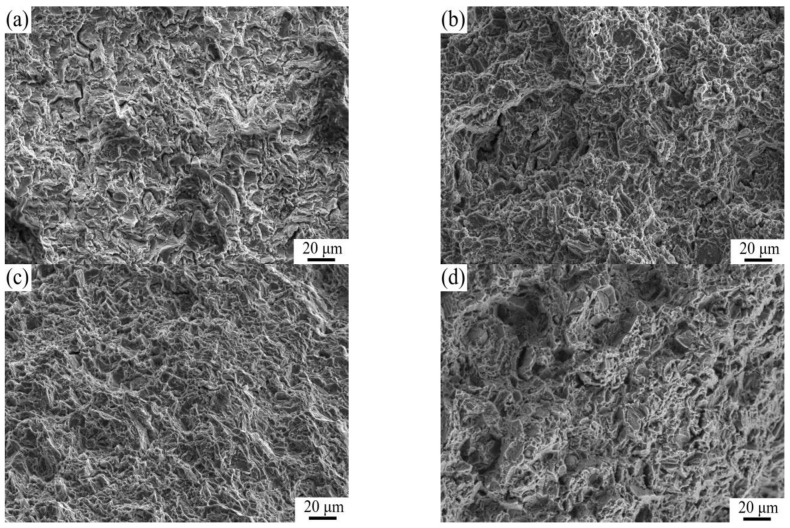
Tensile fracture image of (**a**) AZ31, (**b**) 0.5 wt.%TC4/AZ31, (**c**) 1 wt.%TC4/AZ31, and (**d**) 1.5 wt.%TC4/AZ31 as-extruded composites.

Figure 13a shows the tensile fracture image of the AZ31 alloy; the fracture shows cleavage and quasi-cleavage, and there are some cracks and a few dimples. The fracture mechanism is a mainly brittle fracture; this is due to the HCP structure of the magnesium matrix, and its plasticity is poor [42]. With the increase in nano-TC4 content, as shown in Figure 13b,c, the number of dimples increased and the number of cracks decreased and became less obvious. The increase in dimples means that the plasticity of the material is improved, and the decrease in cracks means that the crack propagation is delayed or even prevented [43], which also means that the plasticity of the material is improved. This is consistent with the tensile test results of the composite. With the further increase in nano-TC4 content, the fracture of the 1.5 wt.%TC4/AZ31 composite is as shown in Figure 13d. The number of dimples decreased significantly and became less obvious; this is a deterioration in the plasticity of the material, and it is also consistent with the tensile test results.

## 4. Conclusions

As an innovative process in this study, TC4 nanoparticles were dispersed to the surfaces of Mg particles by ultrasonic-assisted stirring dispersion, and then a TC4/AZ31 composite was fabricated by SPS following the hot extrusion method. The microstructure and mechanical properties of extruded TC4/AZ31 composites were studied, and the main conclusions can be drawn as follows.

(1) Ultrasonic-assisted stirring dispersion can disperse TC4 nanoparticles uniformly on the surfaces of AZ31 particles. After SPS followed by hot extrusion, TC4 nanoparticles remain uniformly dispersed in the composite material, and are well combined with the Mg matrix; this confirms that the process is feasible.
(2) Nano-TC4 can refine the microstructure of magnesium alloy. The average grain size refinement of the composite is most obvious when the content of TC4 is 1 wt.%, and the average grain size is reduced from 7.02 µm to 2.23 µm.
(3) With the increase in nano-TC4 content, the UTS and FS of the TC4/AZ31 composites increased first and then decreased. When the content of TC4 is 1 wt.%, the mechanical properties of the composite are the best, and the YS, UTS, and FS are 195 MPa, 306 MPa, and 9.2%, respectively, which are 20.4%, 11.7%, and 21.1% higher than those of the magnesium matrix, respectively.

The limitation of this work is that when the content of nano-TC4 exceeds 1.5 wt.%, it is difficult to ensure its uniform dispersion and avoid the appearance of local aggregates. For future research, the uniform dispersion of nano-powder in the magnesium matrix is a prerequisite for the fabrication of nanoparticle-reinforced magnesium matrix composites, and more research is needed on the dispersion process of nanoparticles in the magnesium matrix.

## Figures and Tables

**Table 1:** Chemical composition of AZ31 alloy (wt.%).

Al	Zn	Mn	Fe	Si	Ni	Mg
3.0035	0.6956	0.3035	0.0029	0.0026	0.0015	Balance

**Table 2:** Tensile properties of different magnesium matrix composites.

Materials	YS (MPa)	UTS (MPa)	FS (%)	Reference
AZ31	162 ± 3.1	274 ± 4.9	7.6 ± 0.4	This work
0.5 wt.%TC4/AZ31	184 ± 4.0	289 ± 5.7	8.6 ± 0.7	This work
1 wt.%TC4/AZ31	195 ± 2.2	306 ± 4.2	9.2 ± 0.6	This work
1.5 wt.%TC4/AZ31	202 ± 5.6	301 ± 7.1	7.0 ± 1.3	This work
3 wt.%Ti/AZ31	244 ± 1.9	287 ± 2.8	6.1 ± 0.2	[30]
6 wt.%Ti/AZ31	255 ± 2.7	304 ± 3.7	6.9 ± 0.3	[30]
9 wt.%Ti/AZ31	264 ± 1.6	294 ± 3.4	8.0 ± 0.6	[30]
2 wt.%Al_2_O_3_/AZ31	165	250	4.5	[33]
0.5 vol.%SiC/AZ91	89	203	7.5	[34]
1 vol.%SiC/AZ91	97	222	8.1	[34]
2 vol.%SiC/AZ91	106	126	0.8	[34]

**Table 3:** The parameter values for calculating the theoretical yield strength.

K (MPa·m1/2)	β	G_m_ (MPa)	B (nm)	ΔT (K)	ΔC (K^−1^)	d_p_ (nm)	σ_m_ (MPa)
0.13	1.25	1.66 × 10^4^	0.321	370	17.1 × 10^−6^	60	162

## Data Availability

Not applicable.
